# Bacterial Communities in Effluents Rich in Phenol and Their Potential in Bioremediation: Kinetic Modeling

**DOI:** 10.3390/ijerph192114222

**Published:** 2022-10-31

**Authors:** Miriam M. Morones-Esquivel, Cynthia M. Núñez-Núñez, José L. Hernández-Mendoza, José B. Proal-Nájera

**Affiliations:** 1 Facultad de Ciencias Forestales y Ambientales, Universidad Juárez del Estado de Durango, Río Papaloapan, Valle del Sur, Durango 34120, Mexico; 2 Ingeniería en Tecnología Ambiental, Universidad Politécnica de Durango, Carretera Durango-México km 9.5, Col. Dolores Hidalgo, Durango 34300, Mexico; 3 Centro de Biotecnología Genómica, Instituto Politécnico Nacional, Boulevard del Maestro s/n, esq. Elías Piña, Col. Narciso Mendoza, Reynosa 88710, Mexico; 4 CIIDIR—Unidad Durango, Instituto Politécnico Nacional, Calle Sigma 119, Fracc. 20 de Noviembre II, Durango 34220, Mexico

**Keywords:** native bacteria, molecular identification, bacterial growth, phenolic waste, COD

## Abstract

Phenol is used in the manufacturing process of phenolic resins from which residues remain that must be sent for confinement. For that reason, in this study, the wastewater of a resin factory was analyzed to isolate the bacteria present, identify them by molecular methods and finally evaluate their impact on bioremediation treatment. A total of 15 bacteria were isolated, of these, eight belong to the genus *Bacillus* spp. All bacteria were individually multiplied and inoculated in clusters in 15 L reactors which were carefully monitored for pH, electrical conductivity, chemical oxygen demand and temperature. The acquired data were analyzed using ANOVA with repeated measurements. The first test revealed that native bacterial communities reduce the phenol content by up to 20% and COD by 49%, which is significant with respect to the reactor not being inoculated with bacteria. Furthermore, when a mathematical model was applied to the reactors, it was shown that the bacteria require an adaptation time of approximately 100 h. A second test where the inoculation was interspersed with the addition of lime as a flocculant showed that, even though the reduction in phenol and COD was lower than in the previous test, the difference between treatments and control is statistically significant (α ≤ 0.05).

## 1. Introduction

The treatment of water contaminated with phenol represents an important challenge since phenol and its derivatives are chemical substances that can be produced industrially or naturally and are potentially dangerous even at low levels [1].

The EPA states that below 2 mg/L of phenol in drinking water has no adverse effects and the FDA has determined that the concentration of phenol in bottled water should not exceed 0.001 mg/L. While in the European Union, the limit of this pollutant in wastewater is 0.5 mg/L for surface water (Law no. 152/2006). In Mexico, Federal Law establishes a maximum limit of 0.1 mg/L for water rights [2].

It is estimated that the world production of phenol is six million tons per year with an upward trend [3] and it is considered harmful due to its severe toxicity and for causing cardiac arrhythmias, kidney diseases, and skin cancer, among others [4]. The treatment of waste products is expensive, and it is important to develop an ecological alternative that is friendly to the environment.

Phenolic compounds are common in wastewater from the resin industry (600–2000 mg/L), petrochemical (2.8–1220 mg/L), refineries (6–500 mg/L), coal (28–3900 mg/L) and in industries that process pulp and paper, pharmaceuticals, plastics and paint (0.1–1600 mg/L) [3,5,6]. Coke oven process wastewater also contains phenol (150–2000 mg/L) and cyanide (0.1–0.6 kg/ton coke) at high levels, well above normal limits [7].

Many processes have been tried to reduce the amount of phenols in water, but over the years, biodegradation has been found to be the simplest, cheapest and easiest process for the large volumes of industrial water that are produced [8].

Biological degradation in wastewater has been evaluated with a large number of microorganisms including bacteria, fungi and algae. Pure strains and microbial consortia, several species have been reported as being of potential use, among them: *Gulosibacter* sp., *Pseudomonas putida* and the fungi *Trichosporon montevideense*, *Paecilomyces variotii* and *Candida tropicalis* [5]. A wide variety of microorganisms have been isolated for the biodegradation of phenolic contaminants from different polluted waters, such as *Achromobacter* sp., *Pseudomonas* sp., *Acinetobacter* sp., *Bacillus* sp., *Gulosibacter* sp., *Arthobacter* sp., *Halomonas* sp., *Acinetobacter* sp. (SA01), *Achromobacter* sp., *Rhodococcus* sp. [9,10,11].

Pseudomonas are the bacteria more commonly used in the biodegradation of phenols, especially *Pseudomonas putida*, for which a high degradation capacity has been reported. In the case of fungi, yeasts are the most abundant in polluted environments, since they are capable of consuming a wide variety of carbon sources through enzymatic mechanisms, for which there are possibilities for them to metabolize phenols. A yeast that is capable of efficiently degrading phenol is *Candida tropicalis* for which it has been reported that it can use the pollutant as the only carbon source [12].

However, research on the microbial treatment of high concentrations of phenolic contaminants in actual industrial effluents is limited. Several authors have extensively reviewed the biodegradation of phenolic compounds [13,14]. However, in recent years, in particular, new biodegrading microorganisms, the mechanism of enzymatic metabolic degradation pathway, cometabolic degradation and co-cultivation of phenolic contaminants and the application of sequential systems of anaerobic-aerobic-oxic bioreactors in degradation are lacking.

Even when the mechanism of inhibition by aromatic compounds has not yet been elucidated, it is well known that low molecular mass phenolics are inhibitory for bacterial growth. Phenol is a weak acid; pH influences the inhibitory effect of weak acids. The undissociated form of a weak acid permeates the cell membrane and dissociates in the cytosol; also, the protons are removed by the plasma membrane ATPase in order to neutralize the decrease in internal pH. Very high concentrations of weak acids may result in acidification of the cytoplasm and cell death [15].

In the literature, it is reported that compounds such as calcium oxide, calcium hydroxide, calcium carbonate, among other sources of calcium, are useful for the primary treatment of wastewater since they allow to regulate the pH, adjust the alkalinity, reduce the presence of solids and organic load [16,17,18].

Among the relevant aspects of these studies is the use of calcium hydroxide for the treatment of wastewater from coffee with concentrations of total solids in a range between 7100 and 18,500 mg/L, optimal doses between 0.4% and 0.6% *w*/*v* and removals from 39.7% in BOD_5_ [17]. Similarly, Sevillano [18] evaluated the use of calcium hydroxide in the treatment of acid wastewater from mining exploitation, finding as an optimal dose of calcium hydroxide of 10 g/L with removals of 52.82% of lead, 99.96% of suspended solids and 99.42% dissolved solids.

Vanerkar et al. [19] evaluated the use of lime in the removal of physicochemical parameters in wastewater from food processing, the evaluations consisted of the use of lime alone and in a mixture with chemical coagulants, the results obtained show that lime alone acts efficiently, the optimal dose of lime found was 200 mg/L with removals greater than 40% in suspended solids and chemical oxygen demand.

Traditionally, the identification and estimation of bacterial populations include seeding in a culture medium [20] and individual identification. However, advances in biotechnology allow studies comparing the sequences of the 16S gene and establishing phylogenetic relationships between these populations [7,21,22,23].

In this research, microorganisms from wastewater produced by a resin factory were isolated and identified by PCR means; later, they were used for biodegradation assays in order to assess their efficiency in phenol degradation in four sequential batch reactors working under different conditions. Biodegradation kinetic modeling was then established, and parameters were compared by Student’s t-statistics.

## 2. Materials and Methods

### 2.1. Sampling

Wastewater samples were collected from a resin factory located in Durango, Durango (24.086944° N, 104.668518° W), Mexico, at two sampling sites in the factory facility: the first one, a 90 m^3^ wastewater storage pit, and the second one from a deposit containing laboratory, percolator filters, and industrial trench wastewater.

In the storage pit, samples were taken at three depth levels: top, middle and bottom of the storage pit, taking three samples from each depth.

In the laboratory, wastewater from the washing of the floors and the production of urea resins is channeled into a filtering system of percolator filters. The second sampling took place at the inlet and exit of the percolator filters as well as at the industrial trench where the water exiting the filters is temporarily stored. Three samples were collected at each of these sampling points.

For every sample, pH (Thermo Scientific Orion Star A211, Waltham, MA, USA) and electrical conductivity (H12550 pH/ORP and EC/TDS/NaCl meter HANNA instruments) were measured according to NMX-AA-093-SCFI-2000; the determination of conductivity is of great importance as it gives an idea of the degree of mineralization of the wastewater. Samples’ chemical oxygen demand (COD) was determined according to HACH Method 8000 (spectrophotometer Hach, model DR2010), and the concentration of phenols was determined according to Mexican norms [24].

### 2.2. Isolation and Identification of Microorganisms

The isolation of bacteria was made by the method of dilution in plaque in triplicate in Luria Bertani Agar (BDBioxon^®^, BD, Franklin Lakes, NJ, USA). The bacteria were incubated at 27 °C for 72 h, the colonies were selected by their morphology and coloration and these were multiplied individually in the Luria Bertani broth (200 rpm, 27 °C, 72 h) for molecular identification.

DNA extraction was performed with UltraClean Soil DNA Isolation Kit according to the supplier’s instructions (MO BIO^®^ laboratories, West Carlsbad, CA, USA. The DNA was run in 1% agarose gel, it was visualized in a Photodocumental Molecular Imagen^®^ Gel DocTM XR (Bio-Rad Laboratories Inc., Hercules, Can, USA). For the identification of bacteria, universal primers (Eurofins MWG Operon, Luisville, KY, USA) were used for the gen 16S ribosomal.

The PCRs were performed in a Bio-Rad Cycler ThermoCycler, the products were parted in agarose gel (0.8%) using 100 bp (Ladder DNA Marker) (100–3000 pb, Axygen, Biosciences, CA, USA). The sequencing reaction of purified PCR products was carried out with the commercial ‘Big Dye^®^ Terminator v 3.1 cycle sequencing’ (Applied Biosystems, Waltham, MA, USA). The sequences were edited with the program Chromas Lite and with the program Laser Gene; the assembly was revised with sequence consensus. The identification was made in the BLAST platform of the NCBI (National Center for Biotechnology Information, www.ncbi.org, accessed on 10 January 2021).

### 2.3. Evaluation of Bioremedation Potential by Isolated Microorganisms

**Figure 1:**
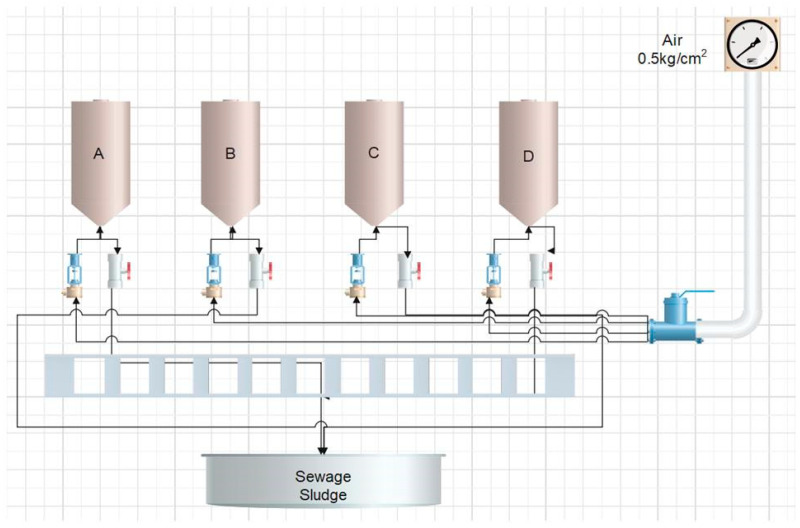
Sequential batch reactors used for biodegradation assays. A: Conditions of reactor A (air 8 days, filtration, inoculum, air 6 days, B: Conditions of reactor B (inoculum and air 13 days, filtration, lime 1 day and filtration), C: conditions of reactor C (lime and air 1 day, filtration, inoculum and air 13 days) D: conditions of reactor D (control).

For the bioremediation assays, four sequential batch reactors (SBR) were built using 6″ PVC tubes, 85 cm in height. The tubes were covered in the extremes and had stainless steel mesh at the bottom. Additionally, two valves were added at the bottom of the reactor body, one valve for the air entrance and a second valve in order to remove the sludge generated during the process and for sampling (Figure 1).

The inoculum was prepared by multiplying the selected native bacteria species individually in the Luria Bertani broth (200 RPM, 27 °C, 72 h) until a concentration of 1 × 10^7^ UFC/mL was reached. A mixture of all the bacteria was made forming an inoculum at a final concentration of 1 × 10^7^ CFU/mL.

#### 2.3.1. First Assay

Base medium (BM) was created by mixing 50% of the water in the retention pit and 50% of the industrial. Each SBR was filled with 15 L of BM and other components, as follows:

For treatment A, BM was added with the bacteria inoculum (1 × 10^7^ CFU/mL), 0.05% molasses and aeration of 0.5 kg/cm^2^; in treatment B, the BM was added with 0.05% molasses and aeration (0.5 kg/cm^2^) but without bacterial inoculum. In treatment C, the mix of BM, bacterial inoculum (1 × 10^7^ CFU/mL), 1.6% calcium oxide, 0.05% molasses, with aeration (0.5 kg/cm^2^) was tested. Finally, Treatment D was taken as a control experiment, so only BM was placed inside SBR.

Samples from every treatment (A, B, C and D) were taken at 0, 24, 120, 216, 312, 408, and 504 h. Electrical conductivity, pH, COD and phenol concentration were recorded. The parameters obtained were subjected to analysis of variance (ANOVA) with repeated measurements, which are usually considered in studies that are longitudinal and measurements are made over time. ANOVA was performed in the software program SAS 9.1 (SAS Institute Inc., Cary, NC, USA), employing as response variables the COD and phenol concentration. Treatments were performed in triplicate.

Removal percentages were calculated according to Equation (1) for Phenol and Equation (2) for COD:

(1) $$\%\mathrm{Ph}=100-\frac{{\left[\mathrm{Phenol}\right]}_{f}\times 100}{{\left[\mathrm{Phenol}\right]}_{i}}\;$$

(2) $$\%\mathrm{COD}=100-\frac{{\mathrm{COD}}_{f\;}\times 100}{{\mathrm{COD}}_{i}}\;$$

where [Phenol]*_i_* and [Phenol]*_f_* correspond to the initial and final concentrations of phenol and COD*_i_* and COD*_f_* represent the initial and final concentrations of COD.

#### 2.3.2. Second Assay

Again, BM was created by mixing 50% of the water in the retention pit and 50% of the industrial. Each SBR was filled with 15 L of BM and other components, as follows (Table 1):

Treatment A consisted of BM, 0.8% of calcium oxide, with aeration of 0.5 kg/cm^2^ and 0.05% of molasses. At 192 h, the solids present in the medium were separated by filtration, then the mixture of bacteria was inoculated (1 × 10^7^ CFU/mL) and aeration conditions were maintained for 144 h.

In treatment B, BM with aeration of 0.5 kg/cm^2^ and 0.05% of molasses, the mixture of bacteria was inoculated (1 × 10^7^ CFU/mL). At 312 h, the solids of the medium were filtered, 0.8% of calcium oxide was added, and at 24 h the solids of the medium were again filtrated.

In treatment C, 0.8% of lime and 0.05% of molasses were added to BM, with an aeration of 0.5 kg/cm^2^. At 24 h, the solids of the medium were filtered, then the mixture of bacteria was inoculated to a concentration of 1 × 10^7^ UFC/mL, to be the same for 312 h.

Treatment D was used as a control and the BM was evaluated.

In all treatments, samples were taken at 0, 72, 120, 168, 216, 264, 312 and 360 h of treatment.

### 2.4. Kinetic Modeling

Based on the unstructured mathematical model of bacterial growth in discontinuous reactors of Pearl and Verlhust [25] and approximations of Gaden for fermentation processes [26], the analysis of degradation kinetics was used according to Quiroga and Sales model as represented by Equation (3) [27] which takes into account the time of adaptation of the microorganisms to the environment where they develop. Equation (3) correlates the rate of substrate consumption with the substrate concentration at each instant and not with biomass production, by means of a second-degree polynomial.

(3) $$\frac{ds}{dt}=K_{2}S^{2}+K_{1}S+K_{0}$$

where *S* is compound concentration and can be calculated according to Equation (4); *S*_0_ is the initial concentration; *p* represents the specified maximum speed of growth of microorganisms calculated through Equation (5), in other words, it assumes that the maximum attainable speed when the term *K*_2_ = 0; *q* represents the concentration of non-biodegradable substrate (Equation (6)); *h* stands for the maximum concentration of invertible substrate in biomass formation, and can be calculated through Equation (7).

(4) $$S=\frac{h\left(S_{0}-q\right)-q\left(S_{0}-h\right)e^{pt}}{\left(S_{0}-q\right)-q\left(S_{0}-h\right)e^{pt}}$$

(5) $$p=\left[\sqrt{K_{1}^{2}-4K_{2}K_{0}}\right]$$

(6) $$q=\frac{\left(-K_{1}+p\right)}{2K_{2}}$$

(7) $$h=\frac{\left(-K_{1}-p\right)}{2K_{2}}$$

These equations were applied to the treatments with the phenol concentration data in the residual water used in the reactors and the degradation time. To solve them, the algorithm of Levenberg–Marquardt for the minimization of squared errors to perform nonlinear regressions was applied through the software Statistical 7.0. The kinetic constants were compared using Student’s *t*-test.

## 3. Results and Discussions

### 3.1. Characterization of Samples and Molecular Identification of Microorganisms

Fifteen (15) bacterial strains were isolated (Table 2), nine correspond to the Phyla Firmicutes, four to the Proteobacteria (of which one is Alfaproteobacteria, another Betaproteobacteria and two more are Gammaproteobacteria). Two belong to Actinobacteria.

Firmicutes are bacteria that have a cover of peptidoglycan on their cell wall that gives them greater resistance to certain environments [28], such as the phenol-rich effluents from where they were isolated. From phyla Firmicutes, *Bacillus subtilis* (AF2) was isolated. These bacteria form endospores that survive in soil with high temperatures and desiccation. There are reports that they can be used in cases of bioremediation of crude soils contaminated with heavy metals and effluents of the textile industry [29]. In addition to extensive use in agriculture as a phytopathogenic or as a plant growth promoter [30] *Paenibacillus lautus* (CF22) has been isolated in oil spills, diesel sludge, in thermoresistant marine environments, such as Antarctica, and in soil, water, rhizosphere and clinical specimens [31,32,33], *B. simplex* (BF9) has antagonistic activity against the phytopathogen fungi *Fusarium* sp. [34] and capable of degrading aromatic compounds in addition to phenol [35]. In addition to bioremediation functions, *B. pumilus* (BE16) and *B. subtilis* have the capacity to produce biosurfactants and use them in reconditioning [36]. On the other hand, *B. frigoritolerans* (BF8) is related to the production of S-methyl thioesters in humans [37] and the bioremediation of agricultural soils [38].

*Micrococcus* sp. (BF7) is an Actinobacteria that has been isolated from a wide variety of environmental conditions including water, soil, human skin and Antarctic ice [39]. Several species of *Micrococcus* spp. can degrade herbicides, chlorinated biphenyls, oils and other complex compounds [40] and *Micrococcus luteus* can produce biosurfactants with the potential for use in bioremediation [36].

From phyla Gammaproteobacteria, *Pseudomonas* sp. (Bf5) was isolated, which has a wide distribution and is associated with the degradation of hydrocarbons and other contaminants. It is reported to degrade phenol, catechol and P-cresol [41,42]. It is also considered to be a plant growth promoter [43]. Of this group, *Citrobacter freundii* (BF21) is also a natural inhabitant of water and soil and it has been reported to degrade phenol [41]. Additionally, the Proteobacteria, *Pseudochrobactrum asaccharolyticum* (AE12) for which there is little information, has been detected in glues and in humans [44] and Long et al. [45] reported their potential application in bioremediation. With regard to Beta Proteobacteria, *Acinetobacter junii* (BE14) has been detected in Cork-making wastewater storage ponds, which have high concentrations of chlorophenols [46]. In this case, the bacteria were multiplied separately and taken individually to a concentration of 1 × 10^7^ CFU/mL and this was the richness of the suspension used in the inoculation of the reactors. In the past, a species from the Acinetobacter genus, *Acinetobacter* EMY, demonstrated the ability for rapid production of a resistant biofilm [47]. In this study, degradation was performed by free cells, in accordance with reports that suggest better phenol degradation by free bacteria that that performed by immobilized populations [48].

The parameters of the phenol-rich effluents evaluated (Table 2) indicate that the samples taken from the retention pit present the highest concentrations of phenol and COD. The composition of the wastewater from the production of resins depends on the manufacturing process (Table 2) and this makes a difference with respect to other published studies that use models of residual water [5,49,50]. Hence, Eiroa et al. [51] reported wastewater from the resin industry with phenol concentrations of 3.3 to 4 mg/L. These values are much less than the ones observed in this study since as mentioned, these are real waters and the concentrations vary according to the working conditions. In all cases, the concentrations of COD and phenol are superior (Table 2) to those allowed by the FDA (0.001 mg/L), the European Union (0.5 mg/L) and Mexican law (0.1 mg/L) [2].

Water color can be indicative of phenol concentrations because when values are high (922 mg/L), residues are pink and as it decreases, they turn to brown and in low concentrations of phenol (15 mg/L) the waters are grey. The values of COD (1613 to 31,940 mg/L) behave in the same way as phenol (15 to 922 mg/L). With regard to electrical conductivity, residual water does not show any major changes between the inlet and outlet of the filters and the retention pit.

### 3.2. Bioremedation Potential by Isolated Microorganisms

#### 3.2.1. First Assay

**Figure 2:**
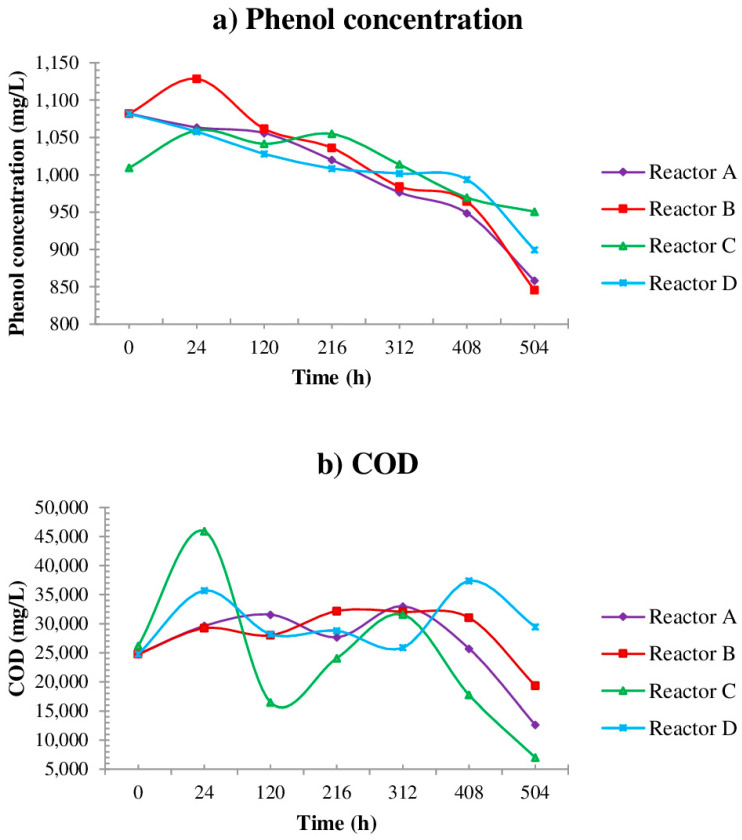
Contaminant degradation by first bioremediation assay.

In this assay it is important to note that the quantity of phenol at the beginning of the test is greater than 1000 mg/L; at the end of the process statistically, there is no significant difference (Pr > F, probability greater than calculated F from Fisher test) between treatments (T reactors), in terms of reduction in Phenol content (Table 3). However, in reactors A and B the reduction was up to 20%, and the reduction kinetics over time (factors time and treatment interaction) present statistically significant differences, meaning the results are substantially different from one another according to statistical testing (Figure 2). As for COD (Table 3), the results show that the treatment in reactor C presented a COD removal of 73.3%, followed by the treatment in reactor A with 49.0% COD removal. Both reactors A and C contained added calcium oxide.

In treatments A, B, and C there is a difference in the kinetics of COD reduction over time (Figure 2b): COD removal of 49.0, 21.9 and 73.3% for reactors A, B, and C, respectively. In the case of reactor D, which corresponds to the control conditions, COD actually increased during the first biodegradation assay. In reactor D, composed of sewage with its native flora, there was a difference (Pr > F) between treatments (T reactors) in terms of reduction in Phenol content (Table 3).

In the case of reactor D, which corresponds to the control, composed of sewage with its native flora, the content of phenol decreased by 16%, but the COD values (Figure 2a,b) increased. From the previous results, it is noted that the microorganisms present a potential use in the treatment of the wastewater of this industry as was observed in reactors A and B. This information appears to be confirmed by the data obtained in reactor D which was not inoculated. The decrease in the values of phenol and COD are important to continue the process of bioremediation of wastewater and to reconcile the products of treatment with other processes such as photocatalysis [52] which requires low concentrations in both parameters.

It is well known that biological treatments are highly efficient for phenol degradation when compared to physicochemical techniques, besides, biological treatments present lower costs. Nevertheless, high phenol concentration in samples could inhibit bacterial activity and result in low phenol degradation [48]. When free bacteria are used for wastewater treatment, certain limitations such as substrate inhibition and sensitivity to environmental conditions are present [53,54].

In the past, Ke et al. [48] used *Bacillus* sp. isolated from activated sludge and subjected to resuscitation and reported a needed lag phase of 20 h in order to achieve good phenol degradation percentages.

The temperature was not taken into account considering previous studies where no remarkable difference was found when operating degradation in different temperatures [14].

#### 3.2.2. Second Essay

For the second trial, the reactors were inoculated with a consortium of all bacteria (grown individually and carried to 1 × 10^7^ CFU/mL) and considering them as a unitary operation. The second modification to the previous trial was formed by the sedimentation of suspended solids with the addition of lime before and/or after the addition of microorganisms as a biological treatment.

Table 4 shows the parameters observed in the second bioremediation assay. The degradation levels important for phenol and COD were obtained with treatment A. This indicates that microorganisms play an important role in the bioremediation of wastewater rich in phenol. This activity is considered practical and economic [8] and among the species that have been reported with potential use is *Pseudomonas putida* [5] which was detected in this study.

**Figure 3:**
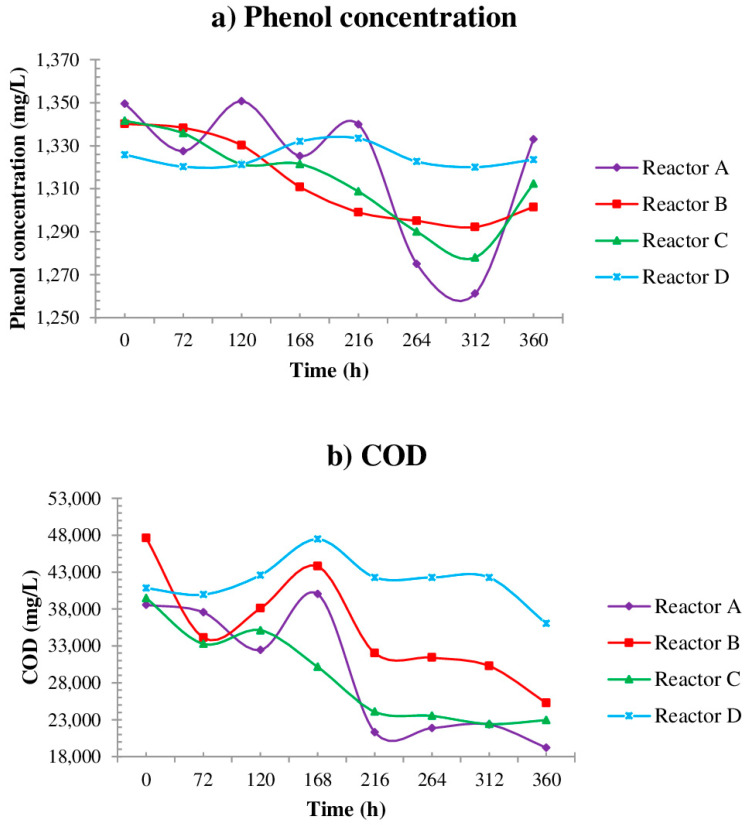
Contaminant degradation by second bioremediation assay.

In this assay, the reduction in phenol represents less than 3% of the initial concentration in the four reactors (Table 4): 1.2% for reactor A, 2.9% for reactor B, 2.2% for reactor C and 0.2% for reactor D (control conditions). COD removal was also greater in reactor A (50.1%), and showed decreasing removal values from reactor B (47.0%) and C (41.8%), being as low as 11.7% in reactor D. In this same test, reactor D, which is a control reactor, as in the previous experiment does not show changes in time. According to the results, the combination of microorganisms plus lime represents the correct conditions to treat wastewater rich in phenolic compounds (Figure 3a,b).

With regard to the effect of high levels of phenol in wastewater, Yoong et al. [55] report that with concentrations of 1300 mg/L or 1200 mg/L of phenol [56], there is a strong inhibitory effect on microbial populations in sewage. The results obtained in this study are consistent with these reports since in the first trial with values slightly higher than 1000 mg/L, the degradation of phenol is 20% and in the second assay with values higher than 1300 mg/L, the degradation of the compound is close to 5%, so it is estimated that native populations are affected by the presence of phenol. For its part, some authors [6] report that it is possible to detoxify mixtures of phenol and form aldehyde with values of 1000 mg/L using advanced oxidation processes (AOP) and aerated biological filters [57].

Hussain et al. [58] used an aerobic sequential batch reactor for the treatment of synthetic wastewater containing phenol (500–3000 mg/L) and found that the acclimatization of activated sludge decreases at concentrations above 2000 mg/L. In addition, they studied the degradation kinetics with the Haldane model and found a maximum growth rate of 0.355 h^−1^, which differs from this study since with the model used, a maximum rate of 0.011 h^−1^ was found for one reactor. In the second test, however, it is necessary to consider that the real water contains not only the phenol but also other substances that can affect the process.

Perales et al. [59], reported the biodegradation kinetics of linear alkylbenzene sulfonates (C_18_H_29_NaSO_3_) with an initial concentration of 20 mg/L in seawater and found a maximum growth rate of 0.226 d^−1^, very similar to that found in this study (0.011 h^−1^ = 0.264 d^−1^) using the Quiroga–Sales model and conclude that the biodegradation of this chemical is slower in seawater.

It has been reported in the past that, under stress conditions, some microorganisms enter a viable but non-culturable state [48], so high phenol concentration could delay microorganism development, causing a period of time with low phenol degradation in experiments as shown in the first 72 h in Figure 3.

Variations in phenol degradation rate can be explained when considering an evolving bacterial community, with different adaptation times to high phenol concentration in the wastewater [60].

The treatment of phenolic waters from the olive industry with an initial COD of 42,250 mg/L when treated with *Bacillus pumillus*, up to 52% reduction of these values was achieved [61]. These results are similar to the ones found in this work, where up to a 50% decrease in COD was reached.

Regarding the importance of the addition of lime in the treatments, there is a determinant effect on the decrease in COD (>70%). Cajigas et al. [62], observed that the maximum efficiency values are associated with the use of sodium bicarbonate and lime. In this study, the use of lime showed a decrease of 73.3% in the COD and it did not significantly influence the pH of the reactors (Table 3).

One of the qualities for which hydrated lime was integrated into this work is due to its high specific surface and its internal cavities that allow it to absorb large amounts of organic compounds [63]. As noted in these trials, their involvement combined with bacteria has synergistic effects and water can be treated more efficiently.

Regarding response variables COD and phenol, the analysis of variance (Table 3) shows significant differences between treatments. An extra parameter that was recorded is the ambient temperature and the statistical analyses did not allow for detecting an impact in the treatments with a significance level of 0.5 (α ≤ 0.05).

### 3.3. Biodegradation Kinetics

**Figure 4:**
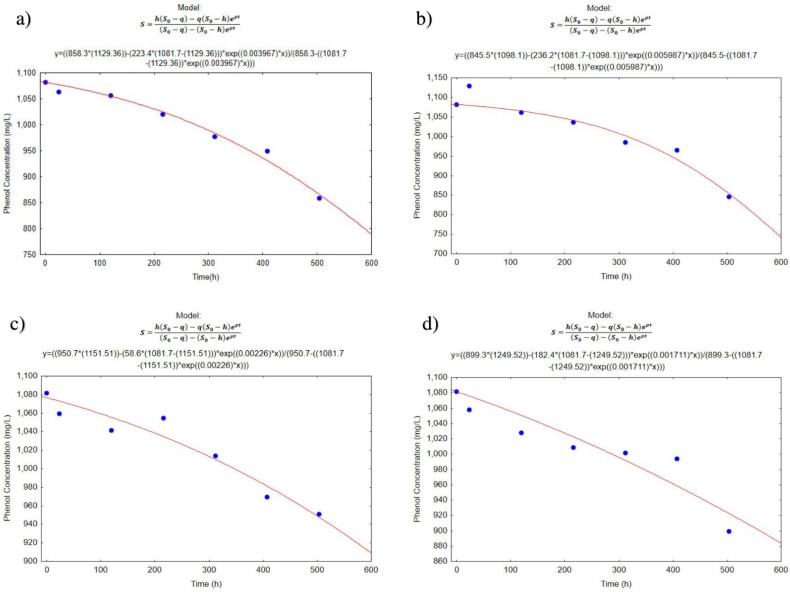
Representation of the experimental (blue points) and theoretical values obtained from the kinetic model of Quiroga-Sales (red line), in the first essay (**a**) Reactor A, (**b**) Reactor B, (**c**) Reactor C and (**d**) Reactor D.

**Figure 5:**
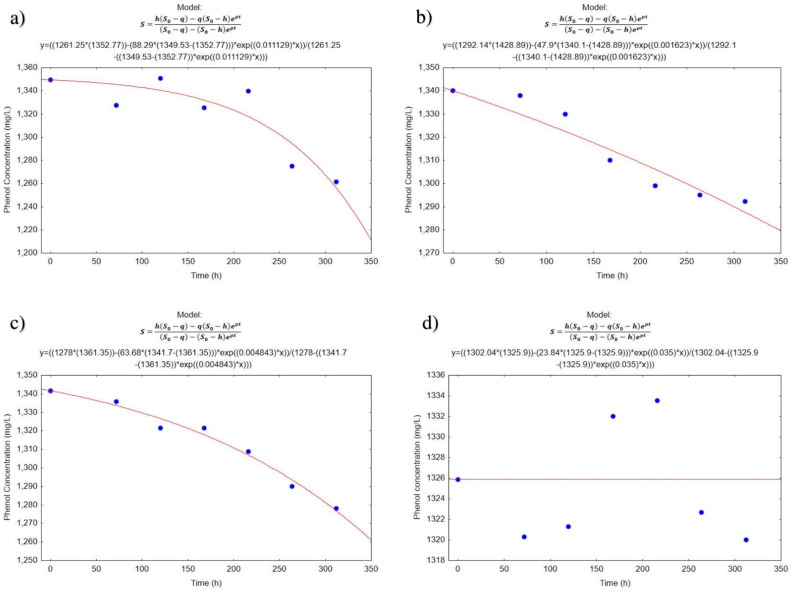
Representation of experimental data (blue points) and theoretical values obtained from the kinetic model of Quiroga-Sales (red line), in the second essay: (**a**) Reactor A, (**b**) Reactor B, (**c**) Reactor C and (**d**) Reactor D.

Figure 4 and Figure 5 present the graphs of the experimental and theoretical values obtained with the Quiroga and Sales model [27]. In reactors A (Figure 4a and Figure 5a) and B (Figure 4b and Figure 5b), it can be estimated that bacterial populations require a period of acclimatization of approximately 100 h, as the decrease in contaminant concentration is too slow during the first days of treatment. With this same analysis, reactor C (Figure 4c and Figure 5c), where the lime was applied shows a decrease in acclimation time and after that period there is an increase in the amount of phenol and then a decrease. In Treatment D (Figure 4d and Figure 5d), it can be observed that the concentrations of phenol are maintained without major changes during the test time. This stability is possibly due to the reactor not being inoculated or maintained with aeration.

The application results of the Levenberg–Marquardt algorithm are shown in Table 5, where *S*_0_, is the concentration of the substrate to t = 0, expressed as a concentration of phenol (mg/L); the *h* represents the sum of the concentration of the organic matter present in the feeding and density of viable microorganisms present in the inoculum (mg/L). In the same equation, *q* represents the concentration of non-biodegradable substrate by the microorganisms and that remain at the end of the experiment (mg/L). Likewise, *p* represents the maximum speed of growth, with units of h^−1^ that correspond to the exponential growth in the first 24 h. The values of *K*_2_ and *K*_0_ are always negative. The values of the term *K*_2_ in general are low and imply that *K*_2_*S*_2_ is a correction factor that includes the growth phase of the microorganisms for their adaptation to the substrate that was given to them to degrade. Although the temperature plays an important role in the kinetics *K* of degradation [27], in this case, the reactors were subjected to very stable temperatures (21 ± 1 °C) so it is dismissed that there is a decisive influence between the reactors. Table 5 shows the calculated parameters for the first experimental assay.

The correlation analysis from which the data was obtained showed a determination coefficient of R (Table 5) which, according to the kinetic model of Quiroga and Sales, microorganisms require long periods of acclimation. The values of the parameter *h* are slightly higher than the initial concentrations of phenol; similar values have been obtained by Romero [64] and Manzano [27] in which h is given by the sum of the initial concentration of the substrate and a term of lesser magnitude is given by the quotient between the initial biomass concentration and the biomass/substrate yield coefficient, corresponding to the maximum amount of substrate available in the medium to form biomass. This same model includes the biomass existing in the medium and can also be degraded by the microorganisms. The value of *p* indicates the speed of biodegradation. With the application of the formula to the results obtained from reactor B, which corresponds to the reactor inoculated with bacteria, enriched with molasses (5%) and 504 h of aeration, a higher rate of degradation is observed, and therefore, the constant *K*_1_ is the largest. The kinetic constants represented by *K*_1_ were compared with the Student’s *t* statistic showing the absence of significant differences between the treatments. By virtue of the foregoing, the second trial was carried out to demonstrate whether the microorganisms play an important role in the degradation of phenol and decrease in COD values in wastewater. For this reason, the second test was carried out, where all the bacteria isolated in the treatments were used and the sedimentation of suspended solids with lime was used as a unitary operation, before and/or after the biological treatment.

Table 6 shows the parameters reported for the second bioremediation assay.

Figure 5 shows that treatment D, to which neither bacteria nor lime were added, was the only one that could not be adjusted (R = 0.4) to the model used. The highest constant and the lowest coefficient R were obtained with treatment A, this could have occurred because it was the one that spent the longest time with lime and this caused fluctuation in concentrations, but also a longer contact time with lime. The lowest constant was obtained with treatment B (Table 6) and treatment C, where lime was added one day and later bacteria were added, has a constant of 0.005 and R = 0.99.

In the comparison of the kinetics of treatments A, B and C, it was obtained that there are significant differences between the treatments, with the constant of treatment A being the highest, which means that the addition of lime for a time and the filtering before of the inoculation of microorganisms allow for estimating the application of these treatments in the bioremediation of real effluents from the production of phenolic resins. Like the analysis of variance, the comparison of the phenol degradation kinetics indicates that the treatments are indeed different, it does influence the moment in which the lime and the microorganisms are added, as well as the time they are in the system, which possibly facilitated the precipitation and the action of the microorganisms.

The kinetic constants represented by *K*_1_ were compared with the Student’s *t* statistic showing the absence of significant differences between the treatments. By virtue of the foregoing, the second trial was carried out to demonstrate whether the microorganisms play an important role in the degradation of phenol and decrease in COD values in wastewater.

### 3.4. Perspectives and Outlook

A combination of treatments seems to be an important tool when treating phenol-rich wastewater. Bar-Niv et al. [47] combined UV-H_2_O_2_ with biological treatment for phenol removal. They found that no phenol degradation was achieved under biological treatment only, but complete removal was reached under a combination of AOP and biological processes.

The simultaneous degradation of phenol and other organic pollutants present in industrial wastewater should be studied, as it may affect the growth of the microbial population. Some researchers suggest that biofilm formation is more effective when degrading phenol and other organic contaminants [48,65,66].

## 4. Conclusions

The wastewater from the resin industry, although containing high levels of phenol (>900 mg/L) and COD (>31,000 mg/L), allows for the establishment of populations of microorganisms that withstand adverse conditions. From these wastewaters, 15 bacteria from the Phyla Firmicutes and Proteobacteria were isolated. These microorganisms, when multiplied and subsequently reintroduced in communities, have an impact on the decrease in phenol levels and COD (>to 20%) in the first trial without being statistically significant. The incorporation of lime in the treatment of residues has a synergistic effect on the reduction in pollutants as detected in the first assay (>70% in COD with respect to the control). Finally, the application of the Quiroga–Sales model was used to estimate the kinetics of waste degradation and to demonstrate that microorganisms require an adaptation period of 100 h before their presence has an impact on the bioremediation of the sewage.

## Figures and Tables

**Table 1:** Second bioremediation assay conditions in each reactor.

Treatments	Reactor A	Reactor B	Reactor C	Reactor D
**Mixture**	50% agarose and 50% industrial wastewater			
**Volume (L)**	15	15	15	15
	Lime (0.8% *w*/*v*)	Inoculum	Lime (0.8% *w*/*v*)	
**Air**	Air (0.5 kg/cm^2^)	Air (0.5 kg/cm^2^)	Air (0.5 kg/cm^2^)	No air
	8 days	13 days	1 day	
	Filtration	Filtration	Filtration	
	Inoculum	Lime (0.8% *w*/*v*)	Inoculum	
	Air	1 day	Air	
	6 days	Filtration	13 days	
**Time**	14 days	14 days	14 days	14 days (360 h)

**Table 2:** Sample characterization and molecular identification of bacterial isolates.

Site	COD (mg/L)	Phenol (mg/L)	Conductivity (mS/cm)	pH	Identification	Phyla
**Surface retention Pit**	31,940 ± 342	922 ± 6.1	2.76 ± 0.4	9.2 ± 0.1	*B. subtillis*	Firmicutes
					*Pseudomonas* sp.	Gammaproteobacteria
					*Micrococcus* sp.	Actinobacteria
					*Brevibacterium frigoritolerans*	Actinobacteria
					*Bacillus simplex*	Firmicutes
					*Citrobacter freundii*	Gammaproteobacteria
					*Paenibacillus lautus*	Firmicutes
**Filter input**	5730 ± 142	142.3 ± 1.4	2.25 ± 0.1	4.7 ± 0.1	*Bacillus safensis*	Firmicutes
**Filter output**	1680 ± 15.3	66.3 ± 16.8	2.62 ± 0.05	7.7 ± 0.1	*Bacillus simplex*	Firmicutes
					*Pseudochrobactrum asaccharolyticum*	Alphaproteobacteria
					*Bacillus pumilus*	Firmicutes
					*Acinetobacter junii*	Betaproteobacteria
					*B. thuringiensis*	Firmicutes
**Industrial Trench**	1613 ± 57.8	15 ± 2.9	0.90 ± 0.01	7.7 ± 0.1	*B. pumilus*	Firmicutes
					*Bacillus* sp.	Firmicutes

**Table 3:** Value of the parameters observed in the first wastewater bioremediation test in the resin industry.

	[Phenol]*_i_* mg/L	[Phenol]*_f_* mg/L	% Ph	COD*_i_* mg/L	COD*_f_* mg/L	% COD	pH	EC mS/cm	T °C
**Reactor A**	1081.7	858.3	20.6 ^a^	24,750	12,611.1	49.0 ^a^	8.1	3.4	22.5
**Reactor B**	1081.7	845.5	21.8 ^a^	24,750	19,322.2	21.9 ^b^	8.3	3.1	20.0
**Reactor C**	1009.3	950.7	5.8 ^a^	26,167	6988.8	73.3 ^c^	10.5	6.3	20.5
**Reactor D**	1081.7	899.3	16.8 ^a^	24,750	29,455.5	−19.0 ^d,^*	8.0	2.4	21.7

[Phenol]*_i_*: Initial phenol concentration, [Phenol]*_f_*: Final phenol concentration, EC = Average electrical conductivity, T: Average temperature. % Ph: Percentage of reduction of phenol, % COD: Percentage of reduction of COD. Different letters after the number indicate statistically significant differences (0.05). Statistically significant differences are calculated by the software program, using Fisher’s test. * COD increased during the first assay.

**Table 4:** Value of the parameters observed in the second bioremediation of wastewater from the resin industry.

	[Phenol]*_i_* mg/L	[Phenol]*_f_* mg/L	% Ph	COD*_i_* mg/L	COD*_f_* mg/L	% COD	pH	EC mS/cm	T °C
**Reactor A**	1349.5	1333.0	1.2 ^a^	38,567	19,211.1	50.2 ^a^	10.07	6.60	25.0
**Reactor B**	1340.0	1301.0	2.9 ^b^	47,633.3	25,266.6	47.0 ^b^	9.06	2.84	24.7
**Reactor C**	1341.6	1312.3	2.2 ^c^	39,466.6	22,966.6	41.8 ^c^	9.07	4.68	24.5
**Reactor D**	1325.8	1323.5	0.2 ^d^	40,816.6	36,033.3	11.7 ^d^	8.66	2.18	24.5

[Phenol]*_i_*: Initial phenol concentration, [Phenol]*_f_*: Final phenol concentration, EC: Average electrical conductivity, T_:_ Average temperature. % Ph: Percentage of reduction of phenol, % COD: Percentage of reduction of COD. Different letters after the number indicate statistically significant difference. All presented values are average values.

**Table 5:** Estimated parameters in the first trial with the application of the Levenberg–Marquardt algorithm.

Parameters	*p* h^−1^	*h* mg/L	*q* mg/L	*S*_0_*-q* mg/L	*K*_0_	*K*_1_	*K*_2_	R
Reactor A	0.004	1129.36	223.4	858	−1.113	0.006	−4.42 × 10^−6^	0.94
Reactor B	0.006	1098.09	236.2	846	−1.805	0.009	−6.96 × 10^−6^	0.90
Reactor C	0.002	1151.00	58.6	951	−0.123	0.002	−1.83 × 10^−6^	0.97
Reactor D	0.002	1249.51	182.4	899	−0.427	0.003	−1.87 × 10^−6^	0.97

*p*: Maximum growth rate, *h*: Sum of the concentration of organic matter present and density of microorganisms in the inoculum, *q*: Non-biodegradable substrate concentration, *S*_0_: Is the substrate concentration at t = 0, *K*_0_*K*_1_*K*_2_: Coefficients of the equation of Quiroga and Sales, R: coefficient of determination.

**Table 6:** Parameters estimated in the second trial with the application of the algorithm Levenberg–Marquardt.

Parameters	*p* h^−1^	*h* mg/L	*q* mg/L	*S*_0_*-q* mg/L	*K*_0_	*K*_1_	*K*_2_	R
Reactor A	0.011	1352.76	88.29	1261	−1.0512	0.013	−8.80 × 10^−6^	0.90
Reactor B	0.002	1428.80	47.98	1292	−0.0805	0.002	−1.18 × 10^−6^	0.95
Reactor C	0.005	1361.34	63.68	1278	−0.3230	0.005	−3.73 × 10^−6^	0.99
Reactor D	0.035	1325.90	23.84	1302	−0.8400	0.036	−2.66 × 10^−5^	0.40

*p*: Maximum growth rate, *h*: Sum of the concentration of organic matter present and density of microorganisms in the inoculum, *q*: Concentration of non-biodegradable substrate, *S*_0_: Is the substrate concentration at t = 0, *K*_0_*K*_1_*K*_2_: Coefficients of the equation of Quiroga and Sales, R: coefficient of determination.

## Data Availability

Not applicable.
